# Cogswell’s Disk Carrier

**Published:** 1886-01

**Authors:** 


### COGGSWELL'S DISK CARRIER.

"Be sure that you go to see the new Coggswell Disk-holder at Codman & Shurtleff's," was the last injunction of Prof. Abbott as we were leaving New York for Boston. We obeyed, saw, and straightway ordered one. It is one of the neatest contrivances imaginable, comprising disk-holder and guard in one instrument. The disks are readily changed, and may be carried with the face out or in, as desired. We have used various devices for disk-holders before, but none that were so complete as is this. We call attention to it, not for the benefit of the manufacturers, but in the interest of the dentists.

